# The Interrelationships of Placental Mammals and the Limits of Phylogenetic Inference

**DOI:** 10.1093/gbe/evv261

**Published:** 2016-01-08

**Authors:** James E. Tarver, Mario dos Reis, Siavash Mirarab, Raymond J. Moran, Sean Parker, Joseph E. O’Reilly, Benjamin L. King, Mary J. O’Connell, Robert J. Asher, Tandy Warnow, Kevin J. Peterson, Philip C.J. Donoghue, Davide Pisani

**Affiliations:** ^1^Department of Biology, The National University of Ireland, Maynooth, Ireland; ^2^School of Earth Sciences, University of Bristol, United Kingdom; ^3^Department of Genetics, Evolution and Environment, University College London, United Kingdom; ^4^School of Biological and Chemical Sciences, Queen Mary University of London, United Kingdom; ^5^Department of Computer Science, University of Texas at Austin; ^6^Department of Electrical and Computer Engineering, University of California, San Diego; ^7^Computational and Molecular Evolutionary Biology Group, School of Biology, Faculty of Life Sciences, University of Leeds; ^8^Mount Desert Island Biological Laboratory, Salisbury Cove, Maine; ^9^Museum of Zoology, University of Cambridge, United Kingdom; ^10^Departments of Bioengineering and Computer Science, University of Illinois at Urbana-Champaign; ^11^Department of Biological Sciences, Dartmouth College, Hanover, New Hampshire; ^12^School of Biological Sciences, University of Bristol, United Kingdom

**Keywords:** placental, phylogeny, mammalian, genome, microRNA, palaeontology

## Abstract

Placental mammals comprise three principal clades: Afrotheria (e.g., elephants and tenrecs), Xenarthra (e.g., armadillos and sloths), and Boreoeutheria (all other placental mammals), the relationships among which are the subject of controversy and a touchstone for debate on the limits of phylogenetic inference. Previous analyses have found support for all three hypotheses, leading some to conclude that this phylogenetic problem might be impossible to resolve due to the compounded effects of incomplete lineage sorting (ILS) and a rapid radiation. Here we show, using a genome scale nucleotide data set, microRNAs, and the reanalysis of the three largest previously published amino acid data sets, that the root of Placentalia lies between Atlantogenata and Boreoeutheria. Although we found evidence for ILS in early placental evolution, we are able to reject previous conclusions that the placental root is a hard polytomy that cannot be resolved. Reanalyses of previous data sets recover Atlantogenata + Boreoeutheria and show that contradictory results are a consequence of poorly fitting evolutionary models; instead, when the evolutionary process is better-modeled, all data sets converge on Atlantogenata. Our Bayesian molecular clock analysis estimates that marsupials diverged from placentals 157–170 Ma, crown Placentalia diverged 86–100 Ma, and crown Atlantogenata diverged 84–97 Ma. Our results are compatible with placental diversification being driven by dispersal rather than vicariance mechanisms, postdating early phases in the protracted opening of the Atlantic Ocean.

## Introduction

**Fig. 1.**
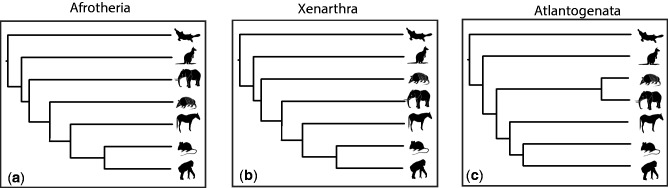
The three principal competing hypotheses for the higher-level relationships among placental mammals, with either (*a*) Afrotheria, (*b*) Xenarthra, or (*c*) Atlantogenata being the sister taxon to all other placentals.

The quest for the root of placental mammal phylogeny has achieved the status of an iconic controversy (Teeling and Hedges 2013), with three principal competing hypotheses that resolve either 1) Xenarthra (e.g., armadillos and sloths; Kriegs et al. 2006; Churakov et al. 2009; O'Leary et al. 2013), 2) Afrotheria (e.g., elephants and tenrecs; Murphy et al. 2001; Asher 2007; Nishihara et al. 2007; Hallstrom and Janke 2010; McCormack et al. 2012; Romiguier et al. 2013), or 3) Atlantogenata (i.e., Xenarthra plus Afrotheria; Murphy et al. 2007; Wildman et al. 2007; Prasad et al. 2008; Meredith et al. 2011; Song et al. 2012; Morgan et al. 2013) as the sister to all other placentals (fig. 1). Previous analyses have found support for all three hypotheses, leading some to conclude that this phylogenetic problem is impossible to resolve (Churakov et al. 2009; Nishihara et al. 2009; Hallstrom and Janke 2010). This has been considered a consequence of incomplete lineage sorting (ILS; Churakov et al. 2009; Nishihara et al. 2009; Hallstrom and Janke 2010; Guo et al. 2012), reflected in large scale gene tree heterogeneity, a result of the apparent rapidity of successive vicariance-driven divergence events associated with the fragmentation of the Pangaean and Gondwanan supercontinents (Murphy et al. 2001; Wildman et al. 2007; Nishihara et al. 2009). Thus, if placental mammals evolved extremely rapidly, then the root of the placental radiation may be theoretically unresolvable, as it was not strictly bifurcating (Nishihara et al. 2009; Hallstrom and Janke 2010) in the first instance. However, it is possible that phylogenetic resolution has been precluded by practical constraints, which include the availability of adequate models of molecular evolution (Morgan et al. 2013), compositional biases, and/or long branch attraction (Romiguier et al. 2013), and computational limitations on the scale of molecular sequence data sets with limited gene and/or taxon sampling (Morgan et al. 2013). Resolution among these three competing hypotheses is essential to understand the evolutionary origin and diversification of placentals, the most phenotypically diverse group of vertebrates, occupying terrestrial, aerial, and aquatic ecological niches, with body sizes spanning several orders of magnitude (Wilson and Reeder 2005) and which were accompanied by both large scale genomic (e.g., transposable elements, Lynch et al. 2015; conserved noncoding RNAs, Mikkelsen et al. 2007) and morphological (e.g., the placenta; Carter and Mess 2007) innovation.

In an attempt to resolve this phylogenetic controversy, we undertook analyses of two genome-scale data sets representing both coding and noncoding regions of the genome: a 21.4 million nucleotide superalignment of 14,631 genes from 36 taxa, and a 16,050 nucleotide superalignment of 239 pre-miRNAs from 39 taxa. In addition, we reanalyzed the data from three recent analyses that obtained results incongruent with those from our protein coding and nonprotein coding data sets (Hallstrom and Janke 2010; O'Leary et al. 2013; Romiguier et al. 2013), and tested the extent to which morphological data can inform mammal phylogenetics using the 4,541 character data set of (O'Leary et al. 2013).

## Materials and Methods

### Phylogenetic Analyses

#### Model Testing

We performed phylogenetic analyses of two nucleotide data sets and three amino acid data sets. The nucleotide data sets were a superalignment of 14,631 protein-coding genes and 36 taxa (totaling 32,116,455), and a superalignment of pre-miRNA sequences comprising 16,050 sites and 42 taxa. The three amino acid data sets were the 11,365 amino acid data set of O'Leary et al. (2013), the AT-rich amino acid data set of Romiguier et al. (2013), and the amino acid data set of Hallstrom and Janke (2010). For all considered data sets Posterior Predictive Analysis (PPA) of biochemical specificity was performed to investigate whether standard, compositionally site-homogeneous, models (e.g., general time reversible [GTR] and Whelan and Goldman [WAG]) provided an adequate fit to the data or whether a more complex (compositionally site–heterogeneous) model (e.g., CAT–GTR; Lartillot and Philippe 2004; Lartillot et al. 2007) was necessary to adequately fit the data. For the nucleotide and microRNA (miRNA) data sets two models were tested, the GTR+G model and CAT–GTR+G. For the amino acid data sets PPA was used to compare the model used in the original studies (Jones, Taylor, and Thorton [JTT]+G [O'Leary et al. 2013]; LG+G [Romiguier et al. 2013]; and WAG+G [Hallstrom and Janke 2010]), against the CAT–GTR+G model. PPA was performed using the serial version of Phylobayes 3.3f (following suggestions from Nicolas Lartillot) using data sets that were subsampled to include a set of approximately 5,000 randomly selected characters. The final number of characters is variable (but comparable) across the different data sets, because of the subsampling strategy we used. However, this is not important as models are compared on the same data sets and not across data sets.

#### General

Total data set size and percentage of missing data is record in table 1. All Bayesian analyses were performed using the CAT–GTR+G model and implemented with the MPI version of the software Phylobayes (Phylobayes MPI 1.5a; Lartillot et al. 2013). For all Phylobayes analyses two chains were run. Burn-in varied and all chains were run until convergence (which was tested using the BPCOMP software, which is part of the Phylobayes suite). Following the Phylobayes manual, chains were considered to have converged on the same solution when the Maxdiff (maximal difference between observed bipartitions) dropped below 0.2. Maximum Likelihood analyses were performed using RAxML (Stamatakis 2006; Stamatakis et al. 2008) under a GTR+G model, and the bootstrap (100 replicates) was used to estimate support.

**Table 1:** Total Size of All Five Data Sets Analyzed and the Percentage of Missing Data in Each

Data Set	Total Sites	% Missing
miRNAs	674,100	22
Nucleotide	770,794,920	39
Hallström and Janke (2010)	7,116,417	21
O'Leary et al. (2013)	522,790	8
Romiguier et al. (2013)	1,065,012	52

#### Nucleotide

The genome alignment of dos Reis et al. (2012), comprising 36 taxa and 14,631 protein-coding genes was used. Codon sequences were aligned using PRANK with no guide tree to minimize bias associated with any guide tree, although we note that alternate alignment software will generate alternate alignments and subsequent analyses should examine whether such alignments affect our results. The first and second codon positions of all genes were concatenated into a single partition (21,410,970 nt). Because of computational limitations, the full data set could only be analyzed using maximum likelihood. We investigated whether the results of our maximum likelihood GTR+G analyses were supported also under CAT–GTR+G, but because a CAT–GTR+G analysis of the entire superalignment is unfeasible, we removed all the constant and parsimony uninformative sites prior to the analysis. We recognize that this is not ideal, as it can introduce biases and this analysis can consequently be considered to have only an exploratory nature. Initial CAT–GTR+G analyses included all the taxa but did not converge. Inspection of the two chains showed that the horse and tree shrew were unstable within Boreoeutheria. As these taxa are irrelevant to investigate the relationships at the root of the placental tree (Boreoeutheria was monophyletic in both chain and with a posterior probability of 1), we repeated analyses excluding these two taxa. This analysis converged on the same topology within 150 generations (with a Maximal Difference between observed bipartitions dropping to zero).

After having run our phylogenetic analyses we investigated whether the data could significantly discriminate between alternative hypotheses of placental relationships. As CAT–GTR+G and GTR+G supported the same tree for the nucleotide data set these analyses were only explicitly performed under maximum likelihood using the GTR+G model. To do so, the three competing hypotheses were fixed and compared using the approximately unbiased (AU)-Test. Sitewise likelihood values were obtained (under each considered hypothesis of placental relationships) using BASEML (Yang 2007), and CONSEL (Shimodaira and Hasegawa 2001) was used to calculate the AU test. Because of computational limitations AU tests was only performed using the superalignment, and not on the 14,631 individual gene alignments constituting our superalignment. For the gene-by-gene analyses a reduced data set of 11,169 genes was used so that every gene had at least one non-placental outgroup, a Xenarthran, Atlantogenatan, and Boreoeutherian present in the alignment so that the tree could not only be rooted but was also informative as to the relationships between these key clades. For each gene we then estimated the likelihood of each considered tree and performed two different analyses. First, we calculated how many genes supported each alternative hypothesis without considering whether the differences in likelihood between compared trees have been significant. This identified the number of genes for which each considered topology is optimal. Subsequently the Akaike Information Criterion (AIC) test was used to determine whether the genes supporting each specific tree topology, supported that topolgy significantly better than the other tree topologies.

#### Incomplete Lineage Sorting

The reduced data set of 11,169 genes from the gene-by-gene analyses (see above) was used to define the set of unbinned gene trees. We also used a statistical binning pipeline (Mirarab, Bayzid, et al. 2014) with support threshold set to 50% to create 2,513 bins of genes (1,373 bins with four genes, 1,139 bins with five genes, and one bin with six genes) and estimated a supergene tree for each bin. ASTRAL version 4.7.6 was run on both sets of inputs: the 11,169 unbinned gene trees, and the 2,513 supergene trees, weighting each supergene tree by size of the corresponding bin (weighted statistical binning; Mirarab, Reaz, et al. 2014; Bayzid et al. 2015). To test for the number of gene trees that supported each hypothesis with support above 50% or 75% threshold, we first restricted each gene tree to branches that have support above the chosen threshold. We then compared each collapsed gene tree against three unresolved trees that represented the three hypotheses. A gene tree can either reject all three hypotheses (i.e., when Xenarthra, Afrotheria, Boreoeutheria, or the branch uniting the three outgroups are rejected), or be indecisive (i.e., be compatible with all three hypotheses; this happens when in the collapsed gene tree, the relationship between Xenarthra, Afrotheria, Boreoeutheria is unresolved), or can support one of the three hypotheses. Thus, five outcomes are possible, and we note the percentage of times each outcome is observed. We also note the percentage of gene trees that support each of the three hypotheses out of those that support just one hypothesis. This produces three estimated probabilities, one for each hypothesis, and we can convert these probabilities to coalescent unit branch lengths by calculating −ln (3/2* (1−*p*)) where *p* is the probability of a hypothesis (Degnan and Rosenberg 2009). For example, for unbinned gene trees, out of 3,495 genes that exclusively supported one the three hypotheses with at least 50% BS, 48.4% of them supported Atlantogenata, which puts the branch length in coalescent units at −ln (3/2* (1–0.484)) = 0.257. Using 75% threshold with unbinned gene trees results in a length of 0.415, and using supergene trees with 50% and 75% threshold result in lengths of 0.135 and 0.192, respectively.

#### microRNA

Small RNA libraries were generated from whole juvenile specimens of Armadillo (*Dasypus novemcinctus*), Rabbit (*Oryctolagus cuniculus*), and Guniea Pig (*Cavia porcellus*) using the Illumina Tru-seq small RNA prep kits. In brief, this process involves taking 1 µg of total RNA and adding 5′- and 3′-adapters, which were then reverse transcribed, barcoded, and amplified using polymerase chain reaction. The sample was run out on a Novex 6% TBE Page gel using electrophoresis allowing size fractionation of the sample. The relevant size fraction will be excised and eluted overnight to increase total product. The eluate will be precipitated using EtOH, glycogen, and sodium acetate for 24 h before being resuspended and submitted for sequencing on a GAIIx sequencer at the University if Bristol Transcriptomics Facility. Total read counts were approximately 22M for Armadillo, approximately 13.5M for Guinea Pig, and approximately 21M for Rabbit, and the data processed using in-house algorithms. These read data were used to verify the mature and star reads and hence the end of the pre sequence, which was used for the pre-mir alignments and have been deposited in miRBase. In addition, BLAST searches were conducted for an additional 42 taxa to identify additional miRNA loci. Orthology for each individual miRNA was checked using both distance and, when possible, syntenic analysis. Each individual pre-miRNA from the 42 taxa analyzed was concatenated into the tetrapod superalignment of Field et al. (2014) and analyzed as a standard superalignment (Tarver et al. 2013; Field et al. 2014; Kenny et al. 2015) comprising 15,590 sites and 42 taxa, using the GTR+G model.

#### Reanalyses

Several recent studies addressed the relationships among the placental mammals finding contradictory results (Hallstrom and Janke 2010; O'Leary et al. 2013; Romiguier et al. 2013). A feature characterizing these studies is the heterogeneity in the choice of the model used for phylogenetic analyses, and the fact that in all cases the substitution model used to analyzed the data was selected in either a subjective way or from a subset of models that did not include well-performing (parameter rich) site-heterogeneous models. Following the results of our PPA (see above), which showed that the models used in the original studies did not fit the data adequately, the three data sets associated with these studies (the 11,365 amino acid data set of O'Leary et al. [2013], the AT-rich amino acid data set of Romiguier et al. [2013], and the amino acid data set of Hallstrom and Janke (2010)) were reanalyzed under the site-heterogeneous CAT–GTR+G model.

### Morphological Data Analysis

O'Leary et al. (2013) recently presented a 4,541 character morphological data set. We tested whether this morphological data set could distinguish between the three alternative hypotheses of placental relationships. As in the case of the nucleotide data set the AU-Test was used (implemented in CONSEL), with character-wise likelihood values estimated in RaXML under the MKv model.

### Molecular Clock Analysis

The 21m nucletotide alignment was used for the molecular clock analysis. This alignment has previously been used (dos Reis et al. 2012), however, the discovery of new fossil material, as well as revised stratigraphy and phylogenetic placement of taxa means that 20 of the 23 calibration points shared between studies had to be revised (table 2). The previously unpublished calibration on node 37 is justified below following best practice guidelines (Parham et al. 2012).

**Table 2:** All 23 Fossil Calibrations Used in This Study

Node		Minimum Soft Bound	Maximum Soft Bound	References
37	Mammalia—Root	201.1^a^	252.23	Herein—see below
38	Theria	156.3^b^	169.6^c^	Benton et al. (2015)
39	Marsupialia	47.6^d^	131.3^c^	Benton et al. (2015)
40	Placentalia	—	164.6^b^	Benton et al. (2015)
42	Xenarthra	47.6^c^	—	Benton et al. (2015)
43	Afrotheria	56.0^b^	—	Benton et al. (2015)
47	Eulipotyphla	61.6^a^	—	Benton et al. (2015)
49	Chiroptera	45.0^a^	58.9	Phillips (2015)
51	Carnivora	37.3^c^	66.0^c^	Benton et al. (2015)
52	Euungulata	62.5	—	dos Reis et al. (2012)
53	Artiodactyla	—	66.0^c^	Benton et al. (2015)
55	Dolphin/Cow	52.4	—	dos Reis et al. (2012)
56	Euarchontoglires	61.6^a^,^c^	—	Benton et al. (2015)
59	Lagomorpha	47.6^c^	66.0^c^	Benton et al. (2015)
60	Rodentia	56.0^c^	66.0^c^	Benton et al. (2015)
61	Guinea Pig/Rat	47.6^c^	59.2^c^	Benton et al. (2015)
63	Muridae	10.4	14.0	dos Reis et al. (2012)
64	Primates	56.0^c^	—	Benton et al. (2015)
65	Strepsirrhini	33.9^c^	56.0^c^	Benton et al. (2015)
67	Anthropoidea	33.9^c^	—	Benton et al. (2015)
68	Catarrhini	24.44^a^	33.9^c^	Benton et al. (2015)
69	Hominidae	11.6^c^	—	Benton et al. (2015)
71	Hominini	6.5^c^	10.0	Benton et al. (2015)

Note.—There are 12 joint (maximum and minimum), two maximum and nine minimum bounds with all maximum and minimum bounds being ‘soft’. Although many of the same nodes are calibrated as in dos Reis et al. (2012), only three of the calibrations are retained with all of the others being revised due to:

^a^Change to a different but existing fossil.

^b^Discovery of a new fossil.

^c^Revision of timescale.

^d^Revision of phylogeny.

### Calibration on Node 37—Mammalia

**Fossil Taxon and Specimen:** *Haramiyavia clemmenseni* (Museum of Comparative Zoology MCZ 7/G95) from the Tait Bjerg Beds, Ørsted Dal Member of the Fleming Fjord Formation with an age corresponding to the Late Triassic (?Norian-Rhaetic) (Jenkins et al. 1997).

**Phylogenetic Justification:** Prior to the discovery of *Haramiyavia clemmenseni*, haramiyids were known from two genera. However, the taxonomic status of these genera was uncertain, and while *H. clemmenseni* exhibited highly specialized dentition it also retained features of the jaw and post-dentary apparatus that indicated a position among stem mammals, cladistically more basal than crown Mammalia, i.e., the clade encompassing monotremes and therians (Jenkins et al. 1997; Zhou et al. 2013). Some recent phylogenetic studies (Zheng et al. 2013; Bi et al. 2014; Krause et al. 2014) have placed Haramiyavia as sister taxon to multituberculates, which are closer to therians than to monotremes and thereby within crown Mammalia. In contrast, other studies argue that the anatomical similarities between haramiyids and multituberculates are convergent (Jenkins et al. 1997; Zhou et al. 2013). We tentatively use Triassic haramiyids as a minimum calibration for Mammalia but are keen to see future, more thorough phylogenetic tests of haramiyid affinities.

**Minimum Age:** 201.1 Ma

**Soft Maximum Age: 252.23 Ma**

**Age Justification:** At present *Haramiyavia clemmenseni* is the oldest known haramiyid from the Tait Bjerg Beds, Ørsted Dal Member of the Fleming Fjord Formation with an age corresponding to the Late Triassic (?Norian-Rhaetic). This stage (Rhaetic) has a minimum bound of 201.3 Ma ± 0.2 Myr (Gradstein et al. 2012) and so the soft minima is set at 201.1 Ma.

**Broader Justification:** *Hadrocodium* and Docodonta (Luo et al. 2002; Meng et al. 2011) are the closest relatives to crown mammals. *Hadrocodium* is from the early Jurassic of Yunnan Province, China (Sinemurian; Luo et al. 2001), and the oldest docodonts are from the Bathonian of Europe, both of which are younger than *Haramiyavia.* More distantly related taxa such as Morganucodontidae, *Sinoconodon*, and *Adelobasileus*, are known from the late Triassic and early Jurassic and are contemporaneous with *Haramiyavia*, implying substantial ghost lineages in many of these taxa, as such a broad prior is used, setting the soft maxima at the PT extinction, dated at the base of the Induan, 252.17 Ma ± 0.06 Myr (Gradstein et al. 2012) and so the soft maxima is set at 252.23 Ma.

The molecular clock analysis was conducted with MCMCTREE v. 4 .8 a (Yang 2007), using the approximate likelihood method (dos Reis and Yang 2011; Thorne et al. 1998) by calculating the maximum-likelihood estimates of the branch lengths, the gradient vector and Hessian matrix, using BASEML, under the HKY+G4 model (Hasegawa et al. 1985; Yang 1994). We then used the Markov chain Monte Carlo algorithm to estimate divergence times on the constrained tree topology with two separate runs being performed. The auto-correlated rates model (Thorne et al. 1998; Rannala and Yang 2007) was used to specify the prior of rates, and we followed (dos Reis et al. 2012) for other parameters, that is; the time unit was 100 Myr; a diffuse gamma prior G(1, 1) was used for the overall substitution rate; a rate drift parameter *σ^2^* was assigned G(1, 1); and the parameters of the birth–death process with species sampling were fixed at *λ* = *μ* = 1 and *ρ* = 0. The alignment was analyzed as a single partition and we conducted 2,000,000 iterations, sampling every 200 a burn-in of 25%, and with both runs being concatenated post burn-in, after thinning down to 10,000 samples per run, to provide the final posterior values.

## Results

### Concatenated 21m Nucleotide Phylogenomic Alignment

**Fig. 2.**
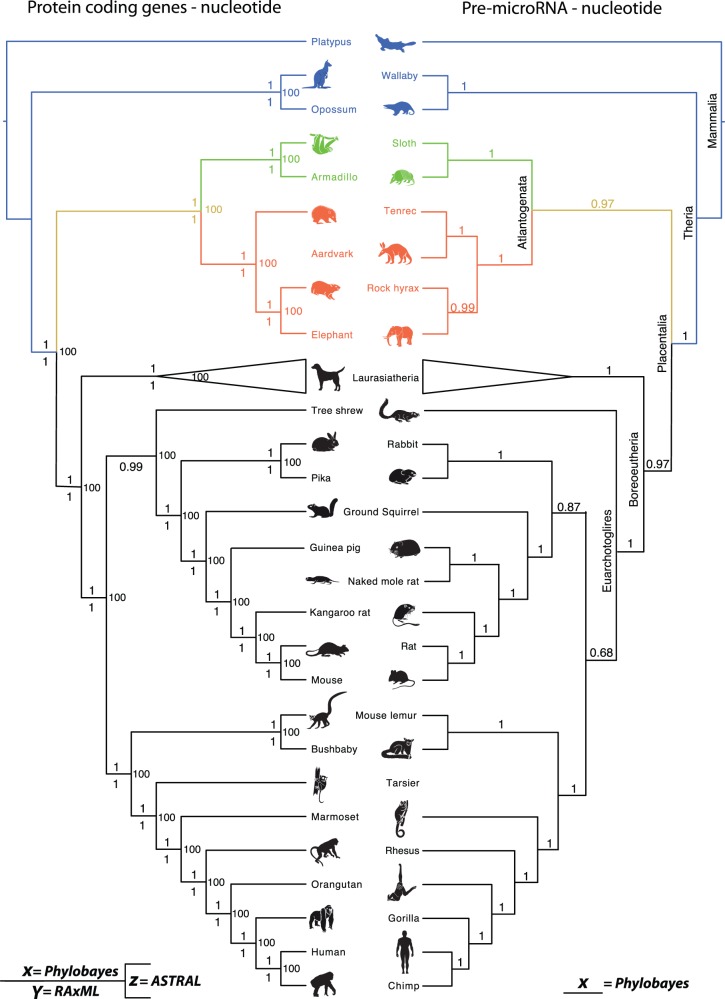
Results from four of the phylogenetic analyses with each one providing support for Atlantogenata as the sister taxon to all other eutherians. (*a*) The 21.4 million whole-genome nucleotide alignment analyzed using Phylobayes (CAT–GTR+G), RAxML and ASTRAL with support values for almost all nodes being either 1 or 100. (*b*) The single concatenated nucleotide alignment for the pre-mir sequences analyzed under GTR+G in Phylobayes. Laurasiatheria is shown collapsed as the interrelationships among the constituent taxa vary between data sets.

A fully resolved phylogeny with 100% support for both a sister group relationship between Afrotheria and Xenarthra (Atlantogenata) and between Atlantogenata and Boreoeutheria (fig. 2, left; supplementary fig. S1, Supplementary Material online) was recovered in the analysis of the 21.4 million nucleotide alignment (first and second nucleotide positions) using a single GTR+G model. Of the 35 internal nodes, 32 were recovered with 100% support. Further analyses were performed using the compositionally site–heterogeneous CAT–GTR+G model, which accommodates among-site amino acid (and nucleotide) compositional heterogeneity. This analysis recovered the same topology with all nodes exhibiting 100% support (fig. 2, left; supplementary fig. S2, Supplementary Material online). Unambiguous statistical support for Atlantogenata was confirmed using the AU test, which assesses the level of support for each topology through a site-by-site analysis of the entire data set. The results of this analysis rejected basal positions for both Afrotheria and Xenarthra (*P* ≤ 0.01) in favor of Atlantogenata (*P* ≥ 0.99) (table 3).

**Table 3:** Results from the Likelihood Tests of the 21.4m Nucleotide Data Set

Topology	GTR+G4, 1st+2nd Sites, 1 Partition			GTR+G4, 1st +2nd+3rd Sites, 1 Gene Per Partition			AIC Test of Significance
	lnL	Delta lnL	AU test	lnL	Delta lnL	%	%
**Atlantogenata**	**−115121891**	**0**	***P* ≤ 0.99**	**−196918173**	**0**	**33.9**	**0.2**
Afrotheria	**−**115123016	1125	*P* **≤** 0.01	**−**196918837	664	33.84	0.12
Xenarthra	**−**115123409	1518	*P* **≤** 0.01	**−**196919286	1113	29.90	0.22
Indecisive						2.30	99.46

**Note**.—The total log likelihoods for the single partition (1st and 2nd sites) were calculated using BASEML under a GTR+G4 model, with the AU test being conducted on these log likelihoods, and showing unequivocal statistical support for Atlantogenata. Additional log likelihoods were then calculated for each individual gene from a reduced dataset of 11,169 genes (see methods) using a GTR+G4 model with all sites included; given the size of this dataset it is computationally impossible to conduct the AU test (as above) although it is clear that Atlantogenata is the most highly supported topology based on the Delta lnL values. Intriguingly, this topology was not supported by a majority of the genes with approximately 30–33% of genes supporting each alternate topology. However, results of the the AIC test of significance show that 99.46% of genes were unable to distinguish between the three competing hypotheses, while the distribution of support for competing topologies reflects the weak phylogenetic signal present in single gene alignments.

Despite strong support for Atlantogenata, we decided to investigate the level of support for each of the three topologies from the individual genes. We therefore removed all of those genes that were unique to individual lineages, that is, Euarchontoglires, Laurasiatheria, Primates etc., or that were not represented by at least one member of Xenarthra, Atlantogenata, Boreoeutheria, and a nonplacental outgroup. This was done so that each individual gene had the potential as to be informative to the placental root, resulting in a reduced data set of 11,169 genes. The number of individual gene trees recovering alternative topologies (albeit not necessarily with high support) is comparable: Atlantogenata (∼33.88%), Afrotheria (∼33.84%), Xenarthra (∼29.9%), and indecisive (∼2.3%) (see table 3). These results could be interpreted to support the prevailing view that the early phylogenetic history of placental mammals was such a rapid radiation that it was not strictly bifurcating. However, 99.4% of the genes fail to discriminate among the competing hypotheses with statistical significance as measured by the AIC test, leaving only 0.2% of genes supporting Atlantogenata, 0.12% supporting Afrotheria, and 0.22% supporting Xenarthra (table 3). Thus, the distribution of support for competing topologies largely reflects the weak phylogenetic signal present in any single gene alignment, rather than suggesting a hard polytomy or very high levels of ILS.

### Coalescent-Based Species Tree Estimation

It is known that concatenation analyses, such as those performed here, can be statistically inconsistent or even positively misleading in the presence of sufficient levels of ILS (Roch and Steel 2015). Thus, we further tested the robustness of our phylogeny through the use of ASTRAL-2 (Mirarab and Warnow 2015), a coalescent-based species tree estimation method that is robust to the presence of ILS (Mirarab, Reaz, et al. 2014). We also explored the use of weighted statistical binning (Mirarab, Bayzid, et al. 2014; Bayzid et al. 2015), a technique designed to improve species tree estimation when gene trees have poor resolution. Thus, we used ASTRAL with and without weighted statistical binning, applied to the same 11,169 genes used in the gene-by-gene analysis described earlier.

**Fig. 3:**
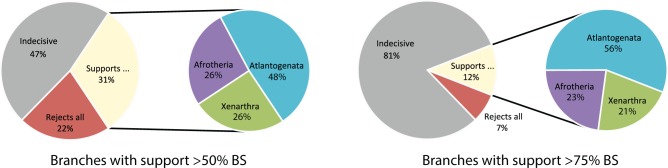
Results from the discordance analysis of the unbinned gene trees with a threshold bootstrap support value of 50% (“left”) and 75% (“right”). These results clearly show that Atlantogenata is the preferred topology, and that much of the incongruence observed across gene trees is due to stochastic errors and not ILS.

In both cases a fully resolved tree with 100% support for Atlantogenata (fig. 2, left; supplementary fig. S3, Supplementary Material online) was returned, supporting the concatenation analysis. After restricting analyses to the set of gene trees with high bootstrap support (50% or 75%) for one of the considered hypotheses, support for Atlantogenata was strengthened (supplementary fig. S4, Supplementary Material online). For example, 48% of the unbinned genes and 42% of the binned supergenes that met the 50% bootstrap support threshold supported Atlantogenata, with almost equal numbers of genes supporting an Afrotheran (26% or 30%) or Xenarthran outgroup (26% or 28%). When the level of bootstrap support necessary for the gene trees to be included in the analyses was increased to 75%, the preference for Atlantogenata further increased to 56% of the unbinned genes, and 45% of the binned supergenes, with corresponding decreases in the levels of support for Afrotheria or Xenarthra (fig. 3). This suggests that some (and perhaps much) of the incongruence observed across the gene trees is the result of stochastic errors in gene tree estimation, not ILS. When restricted to gene tree branches that have bootstrap support above 50% or 75%, the branch length for the Atlantogenata group is between 0.14 and 0.42 coalescent units (depending on the threshold and/or the type of gene trees used; see table 4 and Materials and Methods). Critically, the highest levels of support and longest branch lengths in terms of coalescent units for Atlantogenata are returned when we analyze the data using unbinned gene trees. Our estimated coalescent unit branch lengths point to a short branch, but not an extremely short branch that would violate the hypotheses of a strictly bifurcating tree. These results are largely congruent with concatenation analyses, and suggest that the amount of discordance due to ILS is not sufficient to mislead the concatenation analysis. Thus, although the two analyses are based on data sets of different sizes (11k and 14k genes, respectively), both types of analysis—coalescent-based and concatenation—are highly congruent, and both provide high support for Atlantogenata.

**Table 4:** Shows the Number of Genes, Either Binned or Unbinned Which Support One of Five Outcomes

	Binned (50%)	Unbinned (50%)	Binned (75%)	Unbinned (75%)
Reject all three hypotheses	2994	2418	1673	763
Indecisive	1788	5256	5293	9079
Xenarthra	1751	876	1113	281
Afrotheria	1969	926	1199	303
Atlantogenata	2667	1693	1891	743
Sum of three hypotheses	6387	3495	4203	1327
% Supporting Atlantogenata	0.417566933	0.484406295	0.449916726	0.55990957
Length in coalescent units	0.135075898	0.256971108	0.192220497	0.415309943

Note.—A gene tree can either reject all three hypotheses (i.e., when Xenarthra, Afrotheria, Boreoeutheria, or the branch uniting the three outgroups are rejected), or be indecisive (i.e., be compatible with all three hypotheses; this happens when in the collapsed gene tree, the relationship between Xenarthra, Afrotheria, Boreoeutheria is unresolved), or can support one the three hypotheses. The number of genes that support Atlantogenata is divided by the total number of gene trees that support one of the three hypotheses giving a percentage which can then be used to calculate branch lengths in coalescent units following Degnan and Rosenberg (2009), see Materials and Methods.

### Pre-miRNA Superalignment

In addition to protein-coding genes, we also assembled a concatenated superalignment of 239 noncoding RNA miRNAs consisting of 16,050 nt, which was analyzed under the GTR+G model (see table 5). This miRNA data set provides a second independent molecular data set, that of noncoding RNA genes, to complement protein-coding gene analyses, and these data can be analyzed using the same model based approaches. Such an approach has been shown previously to be suitable in resolving interspecies relationships among reptiles (Field et al. 2014), primates (Kenny et al. 2015), nematodes (Kenny et al. 2015), and drosophilids (Kenny et al. 2015). Our pre-miRNA superalignment recovered a fully resolved tree with an Atlantogenata outgroup exhibiting a posterior probability of 0.97 (fig. 2 right; supplementary fig. S5, Supplementary Material online), in agreement with the protein-coding gene analyses. We again used an AU test to investigate site-by-site support for the three topologies on the entire data set with the results significantly rejecting Afrotheria with *P* = 0.028 (Xenarthra *P* = 0.250; Atlantogenata *P* = 0.795), once more providing support against a hard polytomy.

### Reanalysis of Three Previously Published Data Sets

Given the consistent support in our two data sets for Atlantogenata, we explored why some previous data sets did not find support for this rooting. Amino acid data sets have yielded support for Afrotheria (Hallstrom and Janke 2010) and Xenarthra (O'Leary et al. 2013), and analysis of an AT-rich amino acid data set supported Afrotheria (Romiguier et al. 2013). We focused on model selection and using PPA we showed that the models used in the original studies (WAG2000+G, JTT+G, LG+G, respectively) did not adequately fit the data (see table 5). In contrast, for each of these three data sets, the compositionally site-heterogeneous CAT–GTR+G model was found to be a satisfactory fit to the data. Reanalysis of all three data sets using the CAT–GTR+G model found variable support for Atlantogenata (supplementary figs. S6–S8, Supplementary Material online), and not for the relationships reported in the original studies, undermining their conclusions. Support values for an Atlantogenata root vary considerably between the three reanalyses with values of 1 (Hallstrom and Janke 2010; supplementary fig. S6, Supplementary Material online), 0.79 (O'Leary et al. 2013; supplementary fig. S7, Supplementary Material online), and 0.5 (Romiguier et al. 2013; supplementary fig. S8, Supplementary Material online). While a support value of 50% is uninformative the original paper had a bootstrap support of 100% for Afrotheria. Thus, although this reanalysis does not have high support the use of a better fitting model fundamentally overturned the previous hypothesis, which was itself very highly supported. Likewise, the results of O'Leary et al. (2013), which previously supported Xenarthra, were overturned to support Atlantogenata. Furthermore, these two data sets with the lowest levels of support either contained low numbers of loci (27 nuclear genes) as in O'Leary et al. (2013) or sampled a nonrandom selection of genes, focusing on AT-rich genes as in Romiguier et al. (2013), such approaches are likely to exacerbate phylogenetic artefacts through both compositional and long branch attraction.

**Table 5:** Posterior Predictive Analyses Conducted to Assess the Fit of the Model to the Data

	O'Leary et al. (2013)		Hallström and Janke (2010)		Romiguier et al. (2013)		Nucleotide		miRNAs	
	JTT+G	CAT–GTR+G	WAG2000–G+I	CAT–GTR+G	LG+G	CAT–GTR+G	GTR+G	CAT–GTR+G	GTR+G	CAT–GTR+G
Observed Diversity	3.1336	3.1336	1.8485	1.8485	2.1998	2.1998	3.1998	3.1998	1.3715	1.3715
Posterior Predictive	3.5652	3.1694	2.0711	1.8597	2.3331	2.2086	3.2733	3.2038	1.3297	1.4800
PP Value	0	0.12	0	0.2381	0	0.4367	0	0.3333	0.9588	0.0557

**Note**.—For each of the three previously published data sets, the models used in the original studies, JTT+G, WAG2000+G and LG+G^,^ did not adequately fit the data. In comparison the CAT–GTR+G model, which we used in the reanalyses was an adequate fit to the data. For our nucleotide and miRNAs data sets the CAT–GTR+G model was compared with a GTR+G model, for the nucleotide analysis CAT+GTR+G was found to be the best fitting model, while for the miRNAs data set it was the GTR+G model, in both instances the better fitting model was used.

In addition to their amino acid data set, O'Leary et al. (2013) also used a 4,541 character morphological datamatrix. When this matrix was analyzed using the AU test in RAxML (with a constraint tree to make Afrotheria, Xenarthra, and Atlantogenata monophyletic) the morphological data set was unable to distinguish between the three competing hypotheses (Afrotheria *P* = 0.288, Xenarthra *P* = 0.212, and Atlantogenata *P* = 0.363). Thus, when analyzed in isolation, the morphological data are indecisive concerning the earliest diverging lineage of placental mammals.

### Timing of Placental Radiation

**Fig. 4.**
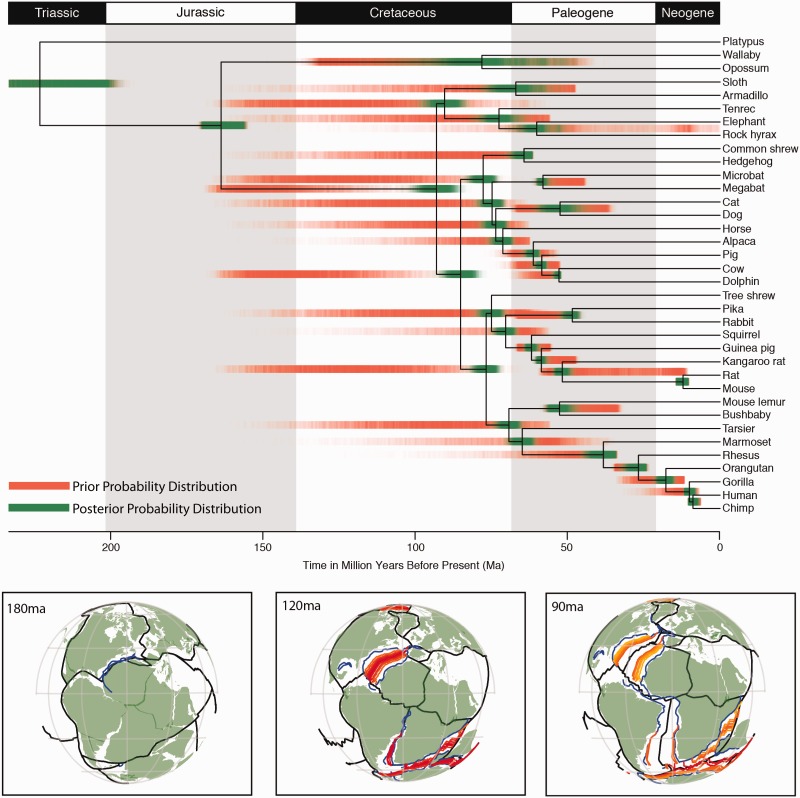
Results from the molecular clock analysis showing the divergence times for placental lineages with all posterior probabilities shown in “green” and overlaid on the joint prior shown in “red,” with both shaded to show values of highest likelihood (see table 6 for the 95% HPD values). Current biogeographic reconstructions for the breakup of Pangea at 180, 120, and 90 Ma, from “left to right,” respectively, with hotter colors (“red”) indicating faster rates of sea floor formation than colder colors (“blue”) based on Seton et al. (2012) and downloadable from http://www.earthbyte.org/Resources/global_plate_model_ESR12.html. Both the Northern and Southern hemisphere continents have separated by 90 Ma, highlighting the role of dispersal, rather than vicariance, for the biogeographic distribution of crown placentals as the breakup of Pangaea predates current molecular clock estimates for the divergence of crown placentals.

We estimate the mean divergence times for crown Theria as 164 Ma (CI = 157–170 Ma), crown Placentalia as 93 Ma (CI = 86–100 Ma), and crown Atlantogenata as 90 Ma (CI = 84–97 Ma) (fig. 4 and table 6). These dates are considerably younger than some studies (Springer et al. 2003; Bininda-Emonds et al. 2007), older than others (O'Leary et al. 2013), and congruent with others still (Hallstrom and Janke 2010; Meredith et al. 2011; dos Reis et al. 2012, 2014). As expected, our revised calibrations, older than those employed by dos Reis et al. (2012, 2014), have the effect of making the posterior ages slightly older (Placentalia and Atlantogenata increase in mean age by 3.1 and 2.5 Myr, respectively), while the 95% CI broadens from 88.3–91.6 to 86.5–99.9 Ma in placentals, and 85.9–89.1 to 83.7–96.5 Ma in Atlantogenata. This broadening in the 95% CI reflects the use of a single data partition, in comparison to dos Reis et al. (2012) in which 20 partitions were used. We estimate diversification of placental orders overlapping the K-Pg mass extinction event at 66 Ma, with all placental orders diversifying between 76 and 51 Ma.

**Table 6:** Prior and Posterior Divergence Times for All Nodes in the Mammal Tree

Node		Marginal Prior^a^			Posterior^b^		
		Mean	95% HPD		Mean	95% HPD	
			Lower	Upper		Lower	Upper
37	Mammalia—Root	226.58	200.99	252.03	223.75	200.47	251.31
38	Theria	163.55	156.45	169.68	163.92	156.67	169.79
39	Marsupialia	95.24	49.77	132.30	78.28	49.11	104.26
40	Placentalia	144.55	115.18	166.80	92.96	86.43	99.91
41	Atlantogenata	119.81	78.70	160.81	90.32	83.73	96.54
42	Xenarthra	80.50	47.46	125.59	67.08	56.62	76.83
43	Afrotheria	90.58	55.92	134.03	72.54	64.85	79.20
44	Paenungulata	49.22	0.12	91.45	60.21	51.45	67.79
45	Boreotheria	135.49	104.09	163.06	85.07	79.93	90.42
46	Laurasiatheria	121.96	84.99	157.18	77.74	73.75	81.96
47	Eulipotyphla	92.65	61.31	132.29	64.35	61.49	67.63
48	Scrotifera	107.66	70.35	146.41	74.82	71.17	78.50
49	Chiroptera	52.07	44.98	58.88	58.23	55.71	60.28
50	Carnivora/Euungulata	95.27	64.18	131.20	73.65	70.18	77.18
51	Carnivora	52.25	37.43	66.15	52.61	45.22	59.70
52	Euungulata	81.43	62.35	110.69	71.35	68.16	74.58
53	Artiodactyla	63.37	55.61	69.78	61.40	59.38	63.40
54	Pig/Cow	59.83	53.00	66.50	58.62	56.92	60.23
55	Dolphin/Cow	56.60	52.21	63.53	52.98	52.10	54.26
56	Euarchontoglires	119.78	84.01	155.50	76.69	72.63	80.60
57	Tree Shrew/Glires	96.61	60.66	136.15	74.93	71.26	78.77
58	Glires	78.48	56.43	115.21	70.34	67.22	73.53
59	Lagomorpha	56.43	47.29	65.59	48.57	46.13	51.65
60	Rodentia	60.89	55.93	65.84	61.97	60.07	63.78
61	Guinea Pig/Rat	53.92	47.79	59.20	58.76	56.91	60.28
62	Kangarro Rat/Rat	36.64	11.75	56.26	51.91	49.14	54.13
63	Muridae	12.18	10.40	13.98	12.21	10.46	13.98
64	Primates	93.18	57.25	130.31	69.27	65.64	72.96
65	Strepsirrhini	44.92	34.04	55.71	52.77	47.91	56.69
66	Haplorrhini	68.64	39.642	108.87	64.96	61.18	68.72
67	Anthropoidea	50.22	33.85	77.91	38.35	33.95	42.77
68	Catarrhini	29.49	24.53	33.97	26.84	24.11	30.32
69	Hominidae	19.04	11.59	28.17	17.85	15.40	20.52
70	Homininae	13.06	6.85	21.03	10.12	8.53	11.45
71	Hominini	8.26	6.51	10.01	8.94	7.51	10.10

^a^The marginal prior was constructed for each node using either the fossil calibrations or from a birth–death process if no calibration was available.

^b^Posterior time estimates for each node based upon the calibrations and the 14k gene data set.

We advocate the use of providing details of not only the combined posterior, but also the marginal prior, which is an analysis run without the sequence data so that the effect of all calibrations can be assessed, as the marginal prior for any node can differ from the original fossil calibration (Warnock et al. 2015). Here we observe that the marginal prior closely approximates (<2 Myr) the calibration points at the majority of nodes, with only four exceptions. The only substantial deviation (>4 Myr) is with the soft minima on node 56 (Euarchontoglires). Such results show that the priors were performing as expected based upon the initial fossil calibrations.

## Discussion

Thus, far from an intractable phylogenetic problem, it is evident that conflicting placental phylogenies have been a consequence of the use of poorly fitting evolutionary models. Evidently, there was some gene tree heterogeneity caused by ILS during placental diversification. However, we can reject the view (Churakov et al. 2009; Hallstrom and Janke 2010) that this was so rampant as to obscure the fundamental relationships among placental mammals. Instead, our results demonstrate that the primary evidence on which such ideas are based, that is, an equal number of genes supporting mutually exclusive topologies, is the consequence of weak signal in single gene alignments rather than the result of ILS alone. As articulated elsewhere (e.g., Gatesy and Baker 2005; Thompson et al. 2012; Pattinson et al. 2015), isolated, historical signal becomes stronger when individual partitions (such as gene alignments) are combined. Thus, we reject the view that the root of the placental mammal tree is an unresolvable polytomy, concluding instead that it is correctly resolved as a fundamental divergence between Atlantogenata and Boreoeutheria.

We do not doubt that evidence of ILS reflects the fact that the initial diversification of placentals was rapid as is observed in our molecular clock analysis, the results of which are comparable to those reported elsewhere (dos Reis et al. 2012, 2014; Meredith et al. 2011). Although the discovery of several recent fossils has led to the calibrations being revised substantially with the minimum ages for the root (Mammalia) and Theria being pushed back 38.2 and 32.3 Myr, respectively, whereas the maxima age for Placentalia was pushed back by 33.1 Myr. Yet, such revisions had only minor changes in the estimated mean age of diversification for Placentalia (+3.1 Myr), Atlantogenata (+2.5 Myr), and Boreoeutheria (+2.6 Myr), however, larger changes were observed for the Mammalia (+38.9 Myr), and Theria (−11.5 Myr) in comparison to the results of dos Reis et al. (2012). These dates support dispersal, rather than vicariance, as the underlying mechanism in placental mammal biogeography as they postdate not only the fragmentation of Pangaea, but also the later splitting of Gondwana due to the opening of the Atlantic Ocean (Seton et al. 2012).

Previous studies (Hedges and Maxson 1996; Wildman et al. 2007; Nishihara et al. 2009) have suggested a clear pattern of biogeographic diversification for placentals into four principle lineages (Afrotheria, Xenarthra, Laurasiatheria, and Euarchontoglires) caused by drift-vicariance, which followed the continental breakup of Pangaea into the northern continent of Laurasia (Laurasiatheria + Euarchontoglires) and a Southern Gondwanan continent (Afrotheria and Xenarthra) in the Jurassic (201.3–145 Ma). This was followed by the later breakup of Gondwana into South America (Xenarthra) and Africa (Afrotheria) due to the opening of the Atlantic during the Cretaceous approximately 110 Ma (Smith et al. 1994; Hay et al. 1999; Milani and Thomaz Filho 2000). Recent analyses of global plate tectonics suggests these dates for the complete breakup of Gondwana into S. America and Africa are too old and that this separation was fully complete by 100 Ma (Torsvik et al. 2009; Seton et al. 2012). However, these dates not only predate our mean divergence time for the divergence of Afrotheria from Xenarthra by approximately 10 Myr, but they also lie outside of the 95% HPD (83.73–96.54 Ma), suggesting dispersal by a group of stem Xenarthrans across the Atlantic. While dispersal across the proto Atlantic Ocean may seem unpalatable, the scale of the Atlantic ocean barrier in the Late Cretaceous (fig. 4) was far less significant than that between Africa and Madagascar which has, nevertheless, witnessed multiple post-Mesozoic dispersal events of placentals, including tenrecs, rodents, primates, and carnivores (Yoder and Nowak 2006). Oceanic dispersal of rodents and primates across the South Atlantic during the Eocene (when the overwater distance between Africa and S. America was wider compared with the Cretaceous) is also uncontroversial (Bond et al. 2015).

With the resolution of the evolutionary relationships among Afrotheria, Boreoutheria and Xenarthra, attention must now turn to resolving the problematic relationships within Laurasiatheria and to understanding of the role of dispersal in effecting placental diversification. The results of both our RAxML and ASTRAL analyses as well as the reanalyses of Hallstrom and Janke (2010) and O'Leary et al. (2013) place the tree shrew as sister taxa to Glires, and the horse in an Euungulata clade, and suggests that classical groupings such as Euarchonta and Ferungulata are not supported. Although such results have been presented before (Meredith et al. 2011) this is an area of significant conflict between previously published studies (Kriegs et al. 2006; Murphy et al. 2007; Nishihara et al. 2009; Hallstrom and Janke 2010; Meredith et al. 2011; McCormack et al. 2012; Nery et al. 2012; Song et al. 2012; Morgan et al. 2013; O'Leary et al. 2013; Romiguier et al. 2013). It is these two rogue taxa (tree shrew and horse) which are the cause of alternate tree topologies, and it is no surprise that the same two taxa were the ones that needed to be removed from our phylobayes analysis as they prevented the runs from converging. In future increased taxonomic sampling of additional perissodactyl lineages, that is, Equidae (donkeys, and zebras), Rhinocerotidae (rhinos), and Tapiridae (tapirs) as well as Scandentia lineages, that is, *Anathana* (Madras treeshrew), *Dendrogale* (Bornean smooth-tailed treeshrew), and *Ptilocercus* (Pen-tailed treeshrew), will lead to increased confidence in the phylogenetic placement of these lineages. While a better understanding for the role of dispersal through not only the late Mesozoic but also the Paleogene (or early Cenozoic) can be achieved through a more precise understanding of the geography including sea-level changes, and not merely the tectonics and biogeography through this interval. In addition the inclusion of fossils within analyses of their living relatives needs to become more widespread, allowing not only greater precision in divergence time estimation through the use of tip dating in molecular clock analyses (Ronquist et al. 2012), but also to better understand the pattern of character acquisition (Patterson 1981), and changes in diversity, either to identify diversification rate shifts (Tarver and Donoghue 2011; Wagner and Estabrook 2014) or broader patterns of biological diversity (Wagner 2000; Tarver et al. 2011; Losos et al. 2013).

The results of our study suggest that other seemingly intractable phylogenetic debates, such as the position of ctenophores, chaetognaths, Acoelomorpha, and the relationships among lophotrochozoans (Dunn et al. 2014), may be solvable by combining genome-scale data sets with realistic models of molecular evolution and rigorous coalescent-based species tree estimation methods.

## Supplementary Material

Supplementary figures S1–S8 are available at *Genome Biology and Evolution* online (http://www.gbe.oxfordjournals.org/).

Supplementary Data

## Note Added in Proof

Whilst this paper was in review following revision, Luo et al. (2015) published a phylogeny which placed haramiyids as stem mammals. In the molecular clock study here haramiyids were considered crown mammals and were tentatively used as a calibration point on the root (node 37). We therefore reran the molecular clock analysis using the much younger Mammalian calibration found in Benton et al. (2015) which places a soft minima at 164.9 Ma and a soft maxima at 201.5 Ma. As expected the root age of Mammalia changes by 40 Myr to become younger, whilst the age of Theria changes by 550,000 years, Marsupialia by 140,000 years and all other nodes by less than 100,000 years. The results of this reanalysis had no material effect on the conclusions of this study.
