# Supplementary material for: The Interrelationships of Placental Mammals and the Limits of Phylogenetic Inference
Source: Genome Biol Evol. 2016 Jan 8;8(2):330–44. doi: 10.1093/gbe/evv261 (PMC4779606; doi:10.1093/gbe/evv261)
Supplement: Supplementary Data [file supp_8_2_330__index.html]

The Interrelationships of Placental Mammals and the Limits of Phylogenetic Inference — Supplementary Data 

# The Interrelationships of Placental Mammals and the Limits of Phylogenetic Inference

## Supplementary Data

files

- Supplementary Data - pdf file
