# Supplementary material for: The Interrelationships of Placental Mammals and the Limits of Phylogenetic Inference
Source: Genome Biol Evol. 2016 Jan 8;8(2):330–44. doi: 10.1093/gbe/evv261 (PMC4779606; doi:10.1093/gbe/evv261)
Supplement: Supplementary Data [file supp_evv261_Tarver_Supp_Figures.pdf]

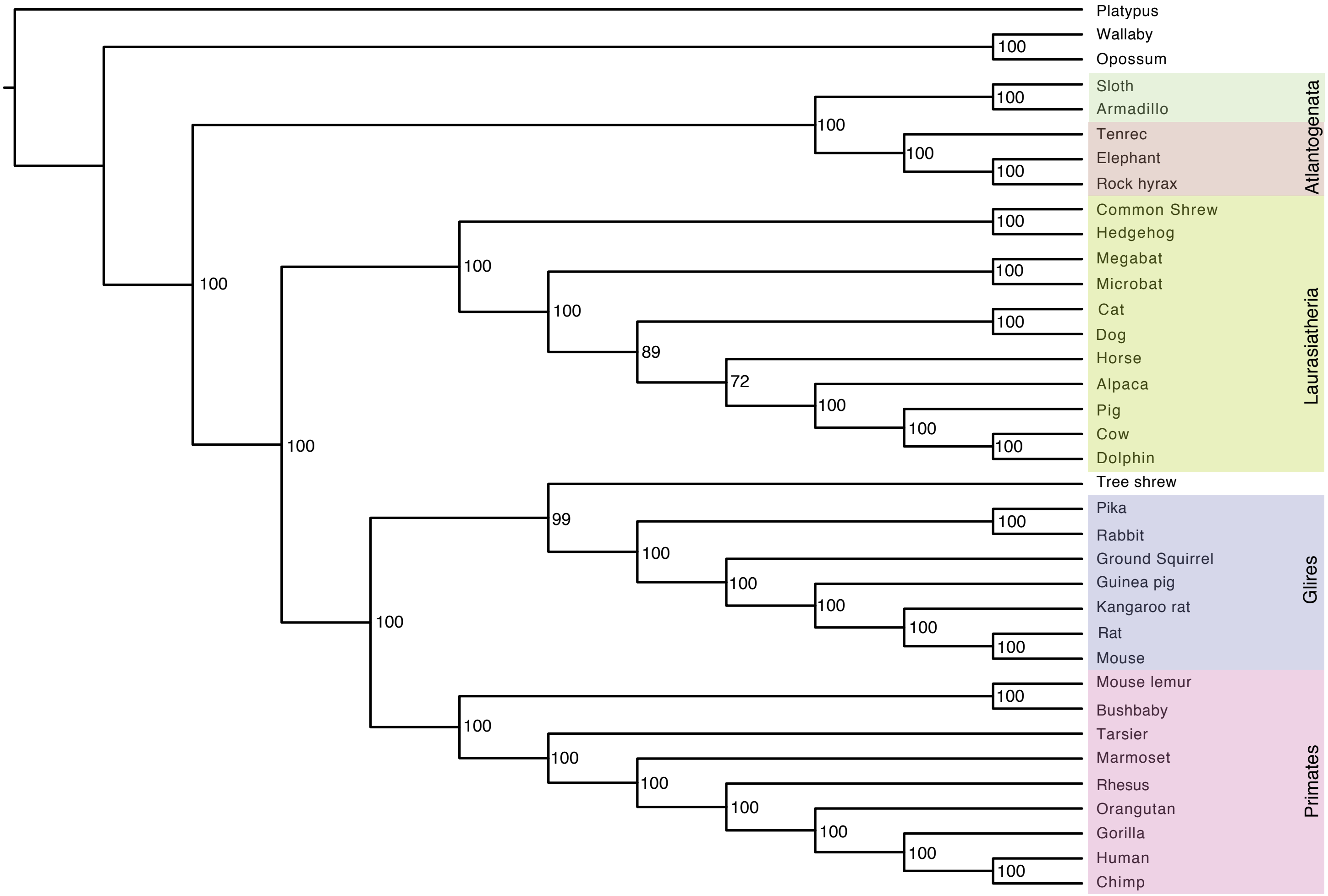

Supplementary Fig. 1: Phylogeny from the 21.4 million nucleotide alignment analysed using RAxML (1st and 2nd positions) under a GTR+G model.

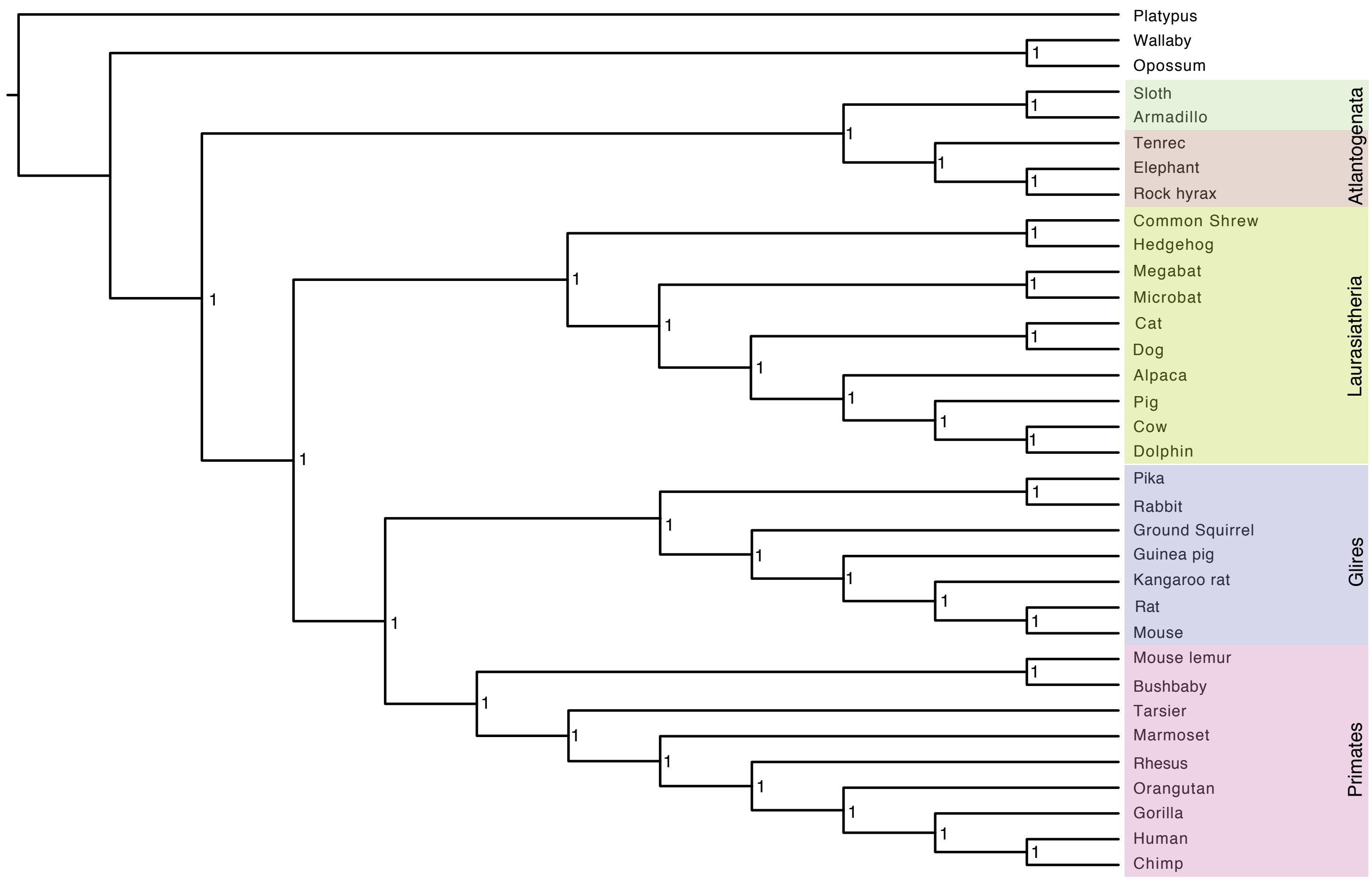

Supplementary Fig. 2: Phylogeny from the reduced nucleotide dataset (parsimony uninformative and constant sites removed) of 4,006,849 nt. In addition both the horse and the tree shrew removed as these taxa prevent the chains from converging. This analysis was run in Phylobayes CAT-GTR+G model.

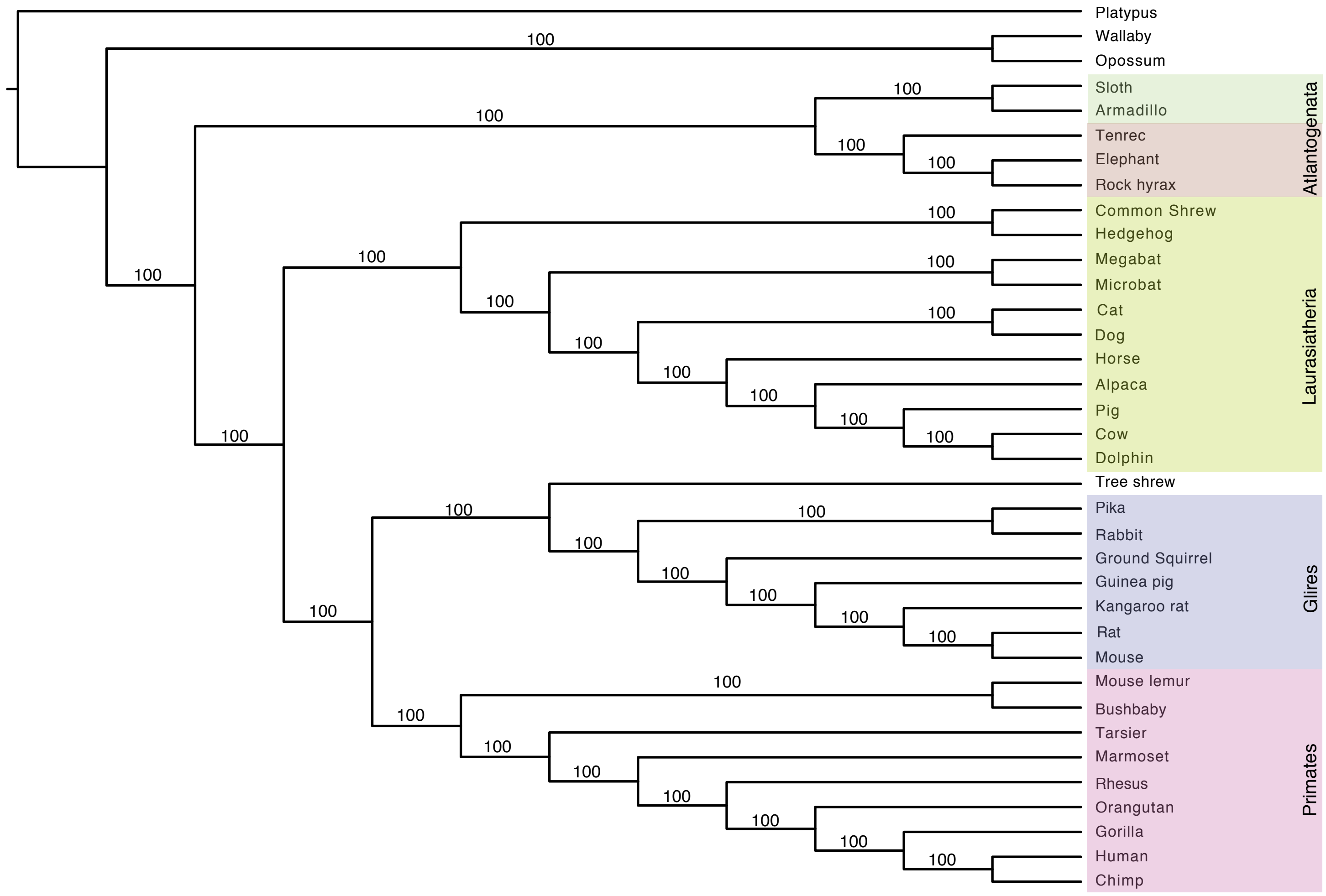

Supplementary Fig. 3: Phylogeny from the unbinned ASTRAL phylogenetic analysis, which supports Atlantogenata and has 100% support for every node.

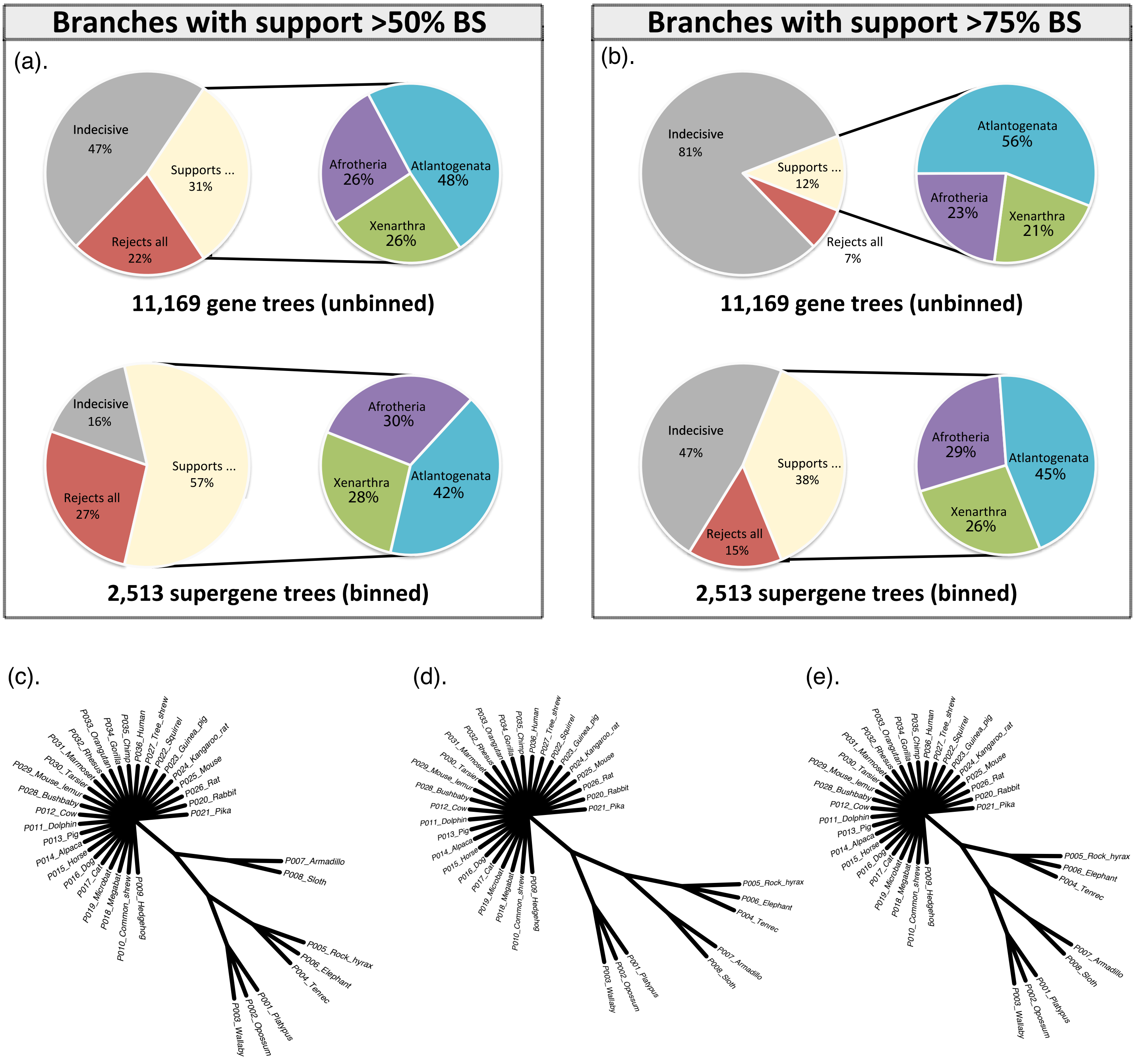

Supplementary Fig. 4: Results from the ASTRAL analysis of the unbinned and binned gene trees with a threshold bootstrap support value of 50% (a) and 75% (b). Three unresolved constraint trees were generated for each of the hypotheses (c) Afrotheria, (d) Atlantogenata, and (e) Xenarthra.

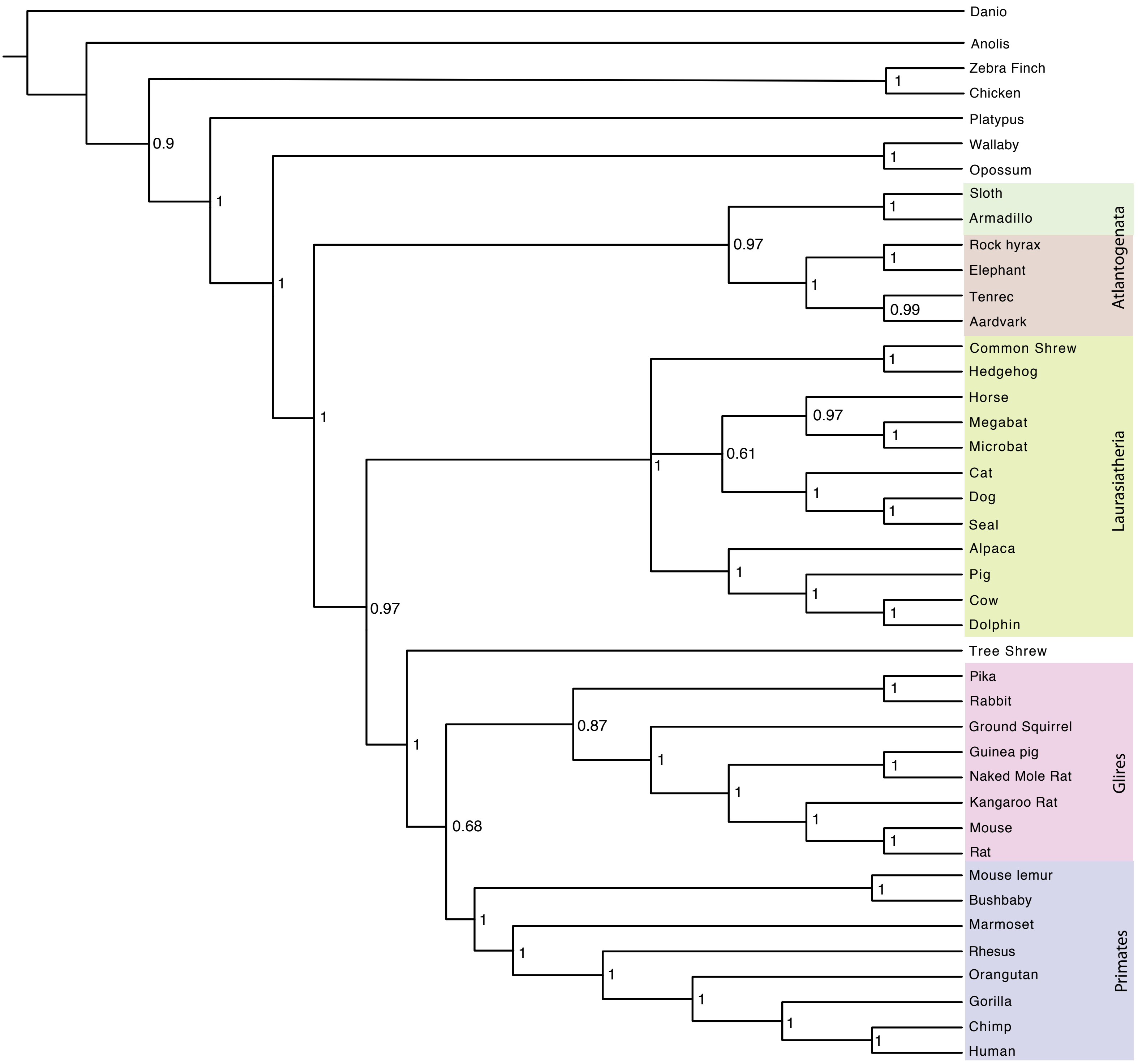

Supplementary Fig. 5: Phylogeny from the 16,050 nucleotide pre-miRNA superalignment analysed in Phylobayes using the GTR+G model.

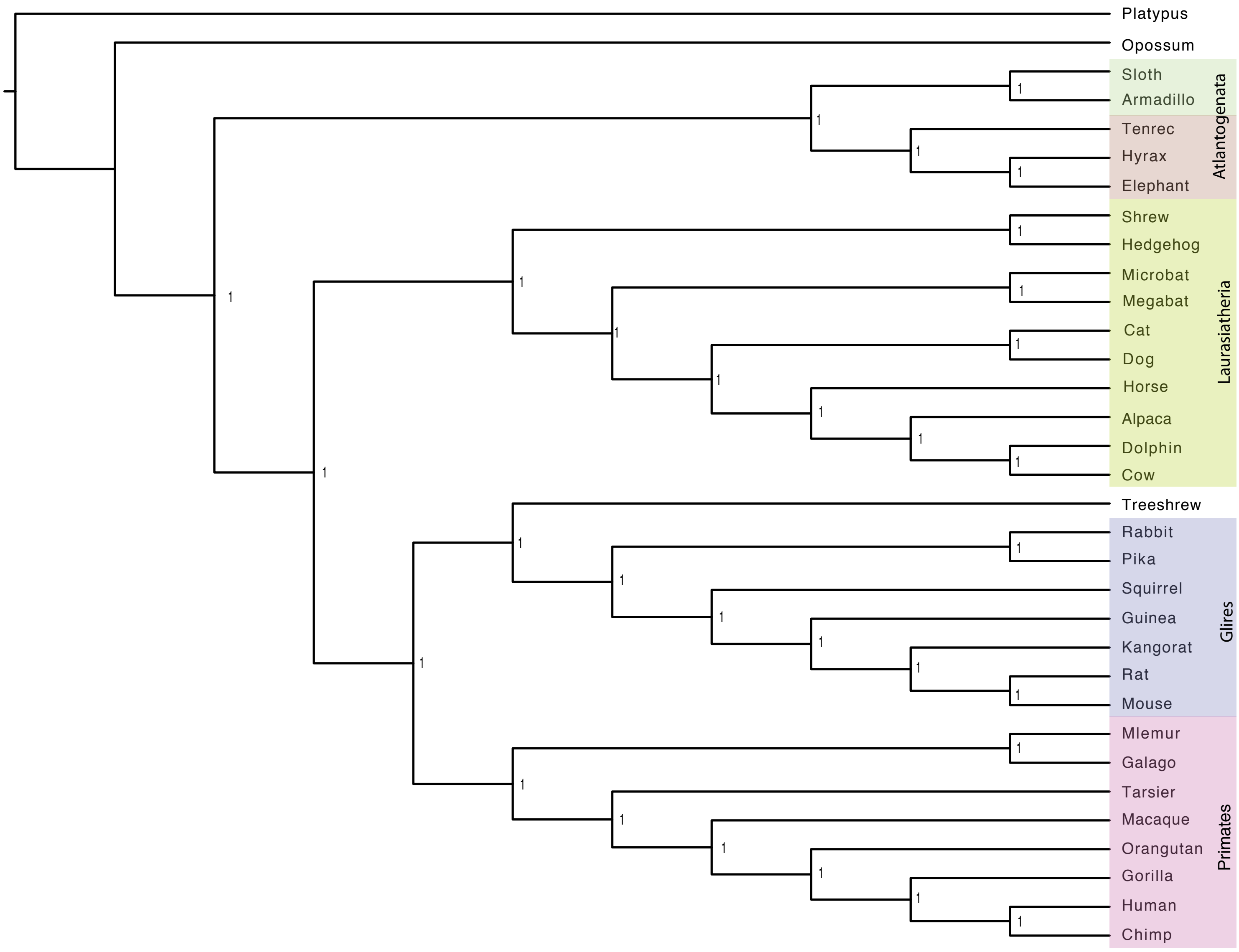

Supplementary Fig. 6: Phylogeny from the reanalysis of the Hallström & Janke (2010) dataset using Phylobayes CAT-GTR+G model.

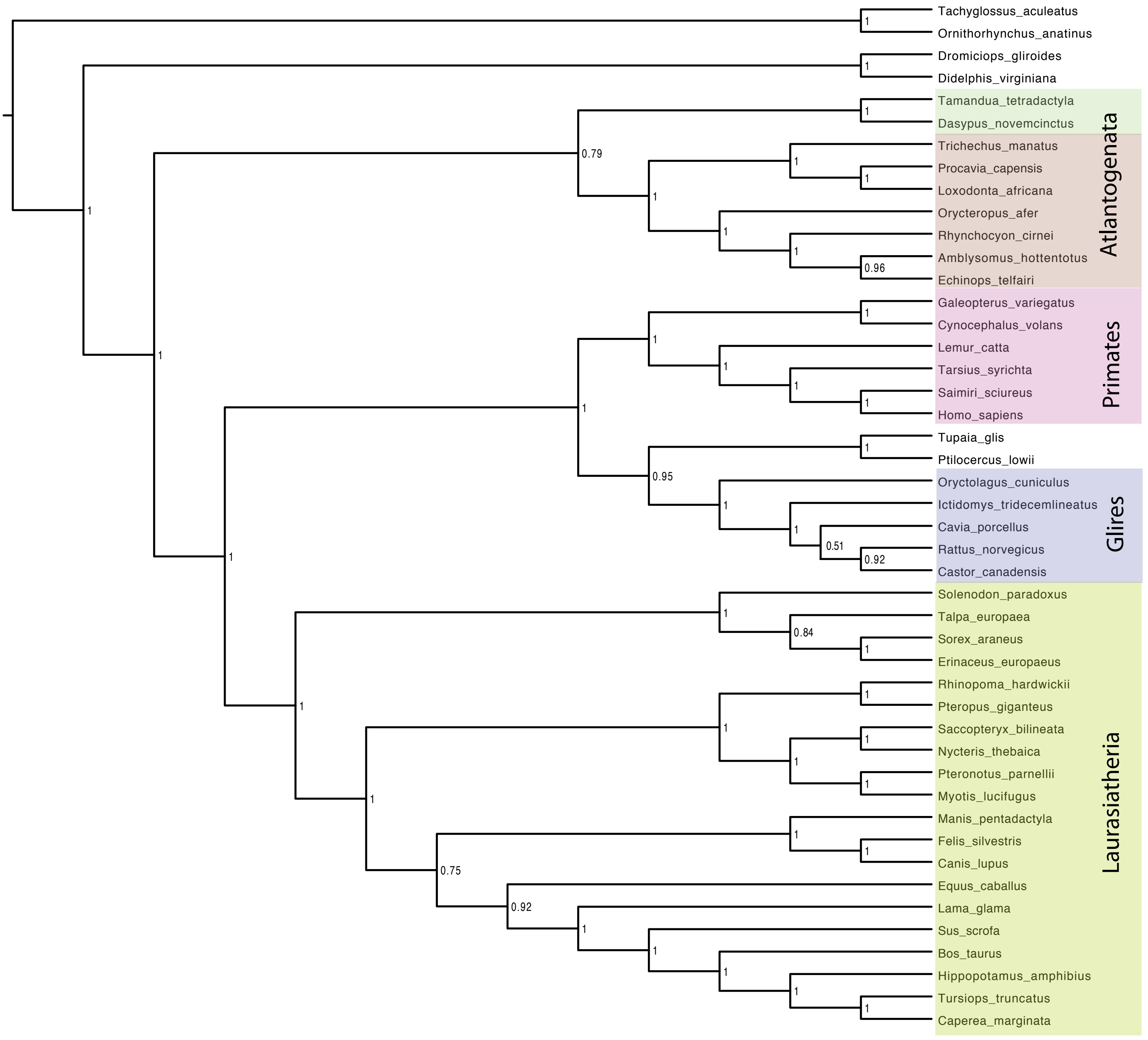

Supplementary Fig. 7: Phylogeny from the reanalysis of the O'Leary et al (2013) dataset using Phylobayes CAT-GTR+G model.

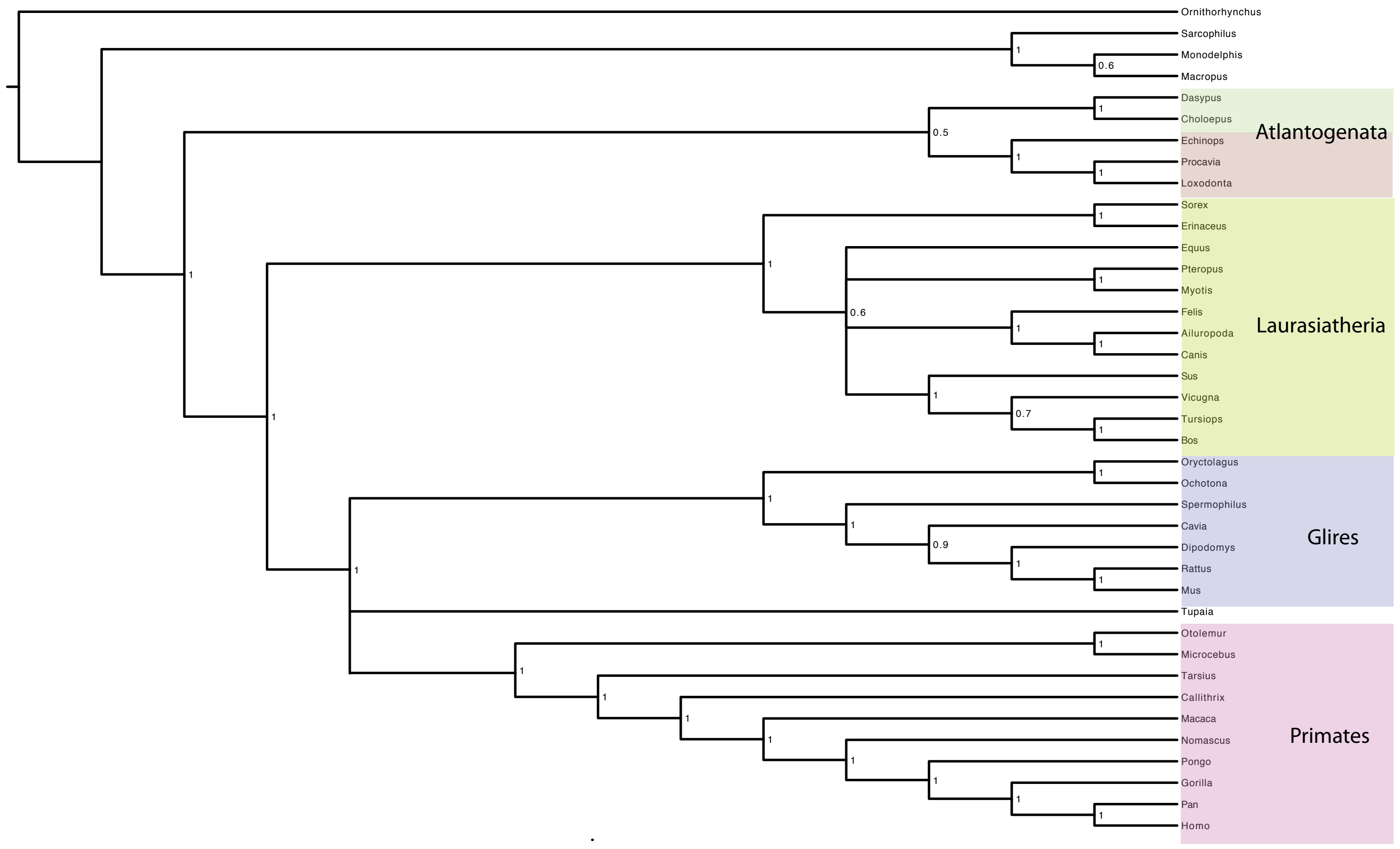

Supplementary Fig. 8: Phylogeny from the reanalysis of the Romiguier et al (2013) high A/T amino acid dataset using Phylobayes under the CAT-GTR+G model.
